# The Relationship between the Spatial and Temporal Evolution of Land Use Function and the Level of Economic and Social Development in the Yangtze River Delta

**DOI:** 10.3390/ijerph20032461

**Published:** 2023-01-30

**Authors:** Rumeng Yin, Xin Li, Bin Fang

**Affiliations:** 1 School of Geographical Sciences, Nanjing Normal University, Nanjing 210023, China; 2 Agricultural College, Yangzhou University, Yangzhou 225009, China; 3 School of Public Administration, Nanjing Agricultural University, Nanjing 210023, China

**Keywords:** land use transition, economic gradient divergence, spatial correlation analysis, sample zones, Yangtze River Delta region

## Abstract

To explore the spatiotemporal evolution characteristics of land use function and its correlation with social and economic development levels, taking the Yangtze River Delta region as an example, we quantified the multifunctional land use in the Yangtze River Delta region from 2000 to 2020 on a 5 km × 5 km grid and analyzed its spatiotemporal evolution characteristics. Each city’s comprehensive measure of economic development used the projection tracing method. Person’s method of interpretation was used for correlation between the spatial and temporal evolution of land use functions and the level of economic development and its coupling association. The study shows that: (1) from 2000 to 2020, the agricultural production function > ecological function > living function > non-agricultural production function in the Yangtze River Delta, but the non-agricultural production and living functions were gradually increasing, while the agricultural production and ecological functions were decreasing. In terms of spatial distribution, the agricultural production function decreases significantly around the built-up area due to the expansion of the built-up area. The non-agricultural production function strengthened around the central city in a network pattern and had a path-locking effect. Topography limits life functions, with high north and low south partially overlapping with non-agricultural production functions. Furthermore, the ecological function was high in the south and low in the north and continues to weaken due to the interference of human activities. (2) The spatiotemporal heterogeneity of different functions generated trade-offs/synergies. The trade-off relationship was prominent in agricultural production and non-farm production function and living function, and non-farm production and living function and ecological function during the study period. Conversely, agricultural production and ecological functions and non-farm production and subsistence functions were generally synergistic. Spatially, there was substantial spatial heterogeneity in the trade-off/synergy relationship between the two functions. (3) There was a clear correlation and spatial coupling between land use function indices and economic development levels in the whole region and sample zones. Still, the dynamic and regional nature of the evolution of land use functions results in sudden changes and jumps in different functions in space. Therefore, in the future integration of the Yangtze River Delta, it is necessary to pay comprehensive attention to the morphology of different land use functions and their synergy/trade-off relationship and to adjust the spatial governance strategy promptly according to the local conditions and the situation.

## 1. Introduction

China’s socio-economic development is strongly constrained by its resources and environment [1], especially the scarcity of land resources and the uneven distribution of their suitability [2], which drive human beings to use land with high intensity and efficiency within a limited space [3]. However, development has also induced many land use problems, such as structural inefficiency [4], high environmental pollution [5], decreasing arable land [6], and health risks [7]. In recent years, land-use transition in the international frontier field of land systems science has become a key and hot topic in land use research. Land use transition refers to the change in the temporal sequence of land use patterns in a country or region, which usually corresponds to a specific socio-economic development stage and provides new perspectives and approaches for comprehensive land use research [8,9]. The key to land use transition research is a deep understanding of land use patterns [10]. According to the Chinese scholar Long Hualou, land use patterns include explicit and implicit forms, with explicit forms manifesting in landscape and operational patterns (e.g., quantity, share or use patterns, type structure). Land use functions are implicit forms containing intrinsic organization and linkages between supply or empirical and demand or normative [11]. Mather’s study of the forest transition hypothesis found that pre-transition primary deforestation had significantly weakened biodiversity. In contrast, post-transition, even with the expansion of forest area, regional biodiversity still struggled to recover, showing that forest functions had been degraded [12,13]. This meant that, in contrast to changes in the spatial pattern of phenotypes, the intrinsic analysis of functional evolution is equally significant [14,15,16]. Scholars understand land use function as the comprehensive provision of products and services that land use systems can supply as direct or indirect carriers in long-term human production and living activities [17,18]. It has become an essential tool for the spatial governance of the land and sustainable socio-economic development.

In the 1960s, developed countries in the West entered the late stage of urbanization and faced many socio-economic transformation problems. Developing rural spatial differentiation from a multifunctional perspective has raised concerns [19,20]. Then landscape multifunctionality, ecosystem services, and land use multifunctionality are also used in many applications [21,22]. There has been extensive and fruitful work on multifunctional land use by scholars nationally and internationally. In terms of research content, from single to diversified, much has revolved around the framework of land use function research [23,24], classification and evaluation system construction [25], spatial and temporal pattern characteristics [26,27], and influencing factors [28]. Concerning research methods, there has been a gradual transition from qualitative to quantitative focus, with typical examples of land grouping or scoring based on subjective behavioral intentions and value measurement of multi-group systems [15,26]. At the research scale, microscopic and refined studies are on the rise, focusing more on macroscopic global pattern revelation in the early stage and, recently, on tiny spatial granularity effects [29]. In addition, with increased application of some remote sensing data (NPP, leaf area index, NDVI) [30,31]. However, there is little essential difference between the research on land use function transformation and land use change. Many transition studies follow the land use transformation analysis paradigm, often analyzing spatial patterns based on functional evaluation and spatial identification. Therefore, the regional, heterogeneous, and changing nature of the land use function transformation process is lacking [11]. In turn, researchers cannot accurately grasp the correspondence with socio-economic development stages, resulting in less reliable research results. The division of the study of economic development stages is also varied, and Western economists represented by Rostow [32], Chinnery [33], Hoffmann [34], Kuznets [35], Northam [36], Friedman [37], and others have conducted a series of explorations on the division of economic development stages from different perspectives. It can mainly include the structuralist view, aggregationist view, and synthesisist view, but structuralism or aggregationism usually use representative indicators to measure, such as GDP per capita of Chinnery’s standard model, but the research scale is relatively large and lacks purchasing power estimation, which will somewhat weaken its ability to conduct horizontal and vertical comparative studies and lack the significance of guidance for policy formulation and implementation. In addition, due to the different strengths and weaknesses of land use functions, the ecological niches of different functions are inconsistent. Moreover, the relationship between multi-functions will be more complex because of the different human needs for multi-functions. For example, they mutually reinforce synergistic relationships or trade-offs between one another [18,31]. If we continue to use extensive temporal correlation analysis, the complex trade-offs/synergies within the space will not be made clear and will weaken the credibility of the findings. We have chosen two methods to overcome these problems: (1) The synergy/trade-off analysis approach in ecology provides lessons for understanding the interlocking relationships of land use functions. Thus, placing land use functions under different levels of economic development can help to analyze differences in trade-offs/synergies. (2) The method of drawing on sample band analysis can fully reflect the spatial and temporal variability. There are many successful applications in global change research [38,39].

In 2019, China’s State Council issued the Outline of the Yangtze River Delta Regional Integrated Development Plan, which elevated the integration of the Yangtze River Delta region to a national strategy to improve the spatial layout of China’s reform and opening up. However, due to natural geographical factors and socio-economic and gradient development policies, the Yangtze River Delta region faces severe problems of socio-economic development and resource factor mismatch [40]. The degree of land use intensification and land use intensity is at a high level, while the quality of agricultural land and ecological land is declining, and the area is shrinking year by year due to the construction land expansion. In addition, regional differences are evident in the Yangtze River Delta region, and the differentiation trend has strengthened. Significant problems exist in the regional division of functions, urban-rural integration, and ecological and green transformation. So, in the context of the integrated development strategy of the Yangtze River Delta and the competition for limited spatial resources, the characteristics of the change of land use functions in the Yangtze River Delta region and its coupled relevance to socio-economic development deserve in-depth exploration. Based on the above, this paper takes the Yangtze River Delta as an example. It simplifies the adequate evaluation of land use functions by overlaying a 5 km × 5 km grid with land class scoring. We used the projection tracing method to measure the regional economic and social development level comprehensively. We used a combination of global correlation analysis and sample strip analysis to analyze the relationship between spatial and temporal changes in land use functions and the level of economic and social development. This paper complements the empirical case study of land use transformation theory. The research process highlights the expression of regional spatial heterogeneity and temporal variability and can identify the problems of sustainable regional development. It is an essential guide for promoting land use control and territorial spatial governance in the Yangtze River Delta region, easing land use conflicts, and achieving synergy between regional resource use and socio-economic development. It also provides an empirical reference for other similar areas.

## 2. Data Sources and Methods

### 2.1. Overview of the Study Area

**Figure 1:**
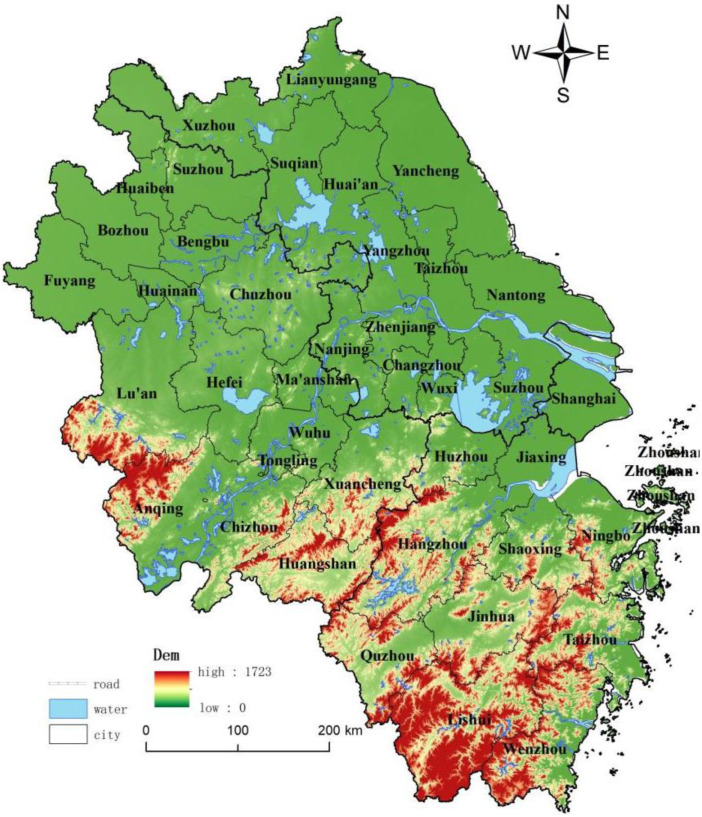
Study area.

The Yangtze River Delta region is the eastern coastal region of mainland China, including three provinces (Jiangsu Province, Zhejiang Province, Anhui Province) and one city (Shanghai). A total of 41 cities, covering an area of 358,000 km^2^, located at latitude 32°34′ N to 29°20′ N and longitude 115°46′ E to 123°25′ E (Figure 1). It is the north-south sample range in China for the IGBP’s 15th standard sample band. The natural environment of the study area varies significantly, and the topography is diverse and zoned. The Yangtze River is the boundary, with the famous Yangtze River Delta Plain to the north, which is gently undulating, and the hilly terrain to the south (e.g., the hilly mountains of southern Anhui and the hilly plains of southwest Zhejiang). A subtropical characterizes the climate to warm temperate transition along the Huaihe-Subei Irrigation Main Canal. The Yangtze River Delta region is one of the regions with the highest level of socio-economic development in China. With 2.2% of China’s land area and 11% of its population, it generates about 20% of its GDP. By the end of 2020, the urbanization rate of the resident population was already higher than 60% and was gradually developing into an influential world-class city cluster. However, the study area faces significant regional economic differences. The more developed regions, with Shanghai as the center of the circle, had formed a networked development pattern, including the Suzhou-Wuxi-Changzhou city cluster, Hangzhou-Jiaxing-Huzhou city cluster, Nanjing metropolitan area, Hefei metropolitan area, Ningbo metro area, and other coastal and riverine areas. At the same time, the northern Anhui, southwestern Zhejiang, and northern Jiangsu regions, located on the periphery of the circle, were relatively underdeveloped. The large gap between the region’s economic and social development stages could be more conducive to regional integration and synergistic development. To solve these problems, narrow the gap in regional development and pursue higher quality development. The Chinese government has formulated and implemented policies and regulations such as the Yangtze River Delta Urban Agglomeration Development Plan and the Outline of Comprehensive Development Plan for the Yangtze River Delta Region. In recent years, the Yangtze River Delta region has implemented an integrated development strategy and made great strides in regional development, with the initial development of a modern industrial system of intelligent manufacturing, new materials, and new energy, a well-developed multinational corporation, and headquarters economy, an enhanced capacity for scientific and technological innovation, and the first signs of industrial clustering. Utilizing urban-rural integration, habitat improvement, and cultural integration, we have completed the regional transportation infrastructure, balanced basic public services, and improved the people’s quality of life. At the same time, the developed industrial economy has also induced many ecological and environmental problems in the Yangtze River Delta. Large pollution discharge and industrial wastewater discharges, resulting in frequent outbreaks of problems such as cyanobacteria in Taihu Lake and pollution of the waters of Hangzhou Bay [41]. Habitat quality has declined, and the expansion of land for construction has triggered a sharp decline in ecological land, particularly in the depletion of wetlands. The dense concentration of heavy chemical companies along the Yangtze River has led to air quality concerns, and cancer villages and endemic diseases have also raised concerns. Since the mismatch between resources, environment, economy, and society hinders the high-quality development of the region [42], there is an urgent need to transform the land use function to solve this dilemma.

### 2.2. Data Sources

The data for this study consisted of the following six main categories. (1) Remote sensing monitoring data of the Yangtze River Delta for 2000, 2010, and 2020 from the Data Center for Resource and Environmental Sciences, Chinese Academy of Sciences (http://www.resdc.cn), accessed on 5 July 2022. In the acquisition of land use status map from Landsat TM/ETM+/OLI remote sensing images, the primary processing steps include image pre-processing and manual visual interpretation. According to the Classification of Land Use Status (GB/T21010-2017), we identify land use types into six categories: arable land, forest land, grassland, water, construction land, and unused land. The overall accuracy of the study area reaches above 80%, and the Kappa coefficient is above 0.7. (2) Socio-economic statistics: The socio-economic data used in this paper come from the Shanghai Statistical Yearbook (2001~2021), Jiangsu Statistical Yearbook (2001~2021), Zhejiang Statistical Yearbook (2001~2021), Anhui Statistical Yearbook (2001~2021), China County Statistical Yearbook (2001~2021), and China City Statistical Yearbook (2001~2021). (3) DEM data were obtained from the geospatial data cloud (http://www.gscloud.cn/ accessed on 29 November 2022) with 90-m resolution. (4) The road data are obtained from the ‘National Earth System Scientific Data Sharing Platform-Yangtze River Delta Scientific Data Center’ (http://www.geodata.cn), accessed on 5 July 2022 and the open-source wiki map OpenStreetMap (OSM), with a spatial resolution of 1:250,000. (5) The data of administrative divisions in the Yangtze River Delta region are obtained from the China National Basic Geographic Information Center (http://ngcc.sbsm.gov.cn), accessed on 5 July 2022. The study considers the adjustment of administrative divisions and the continuity of the above data. Therefore, we choose the latest administrative divisions for the consolidation process.

### 2.3. Study Ideas and Main Methods

**Figure 2:**
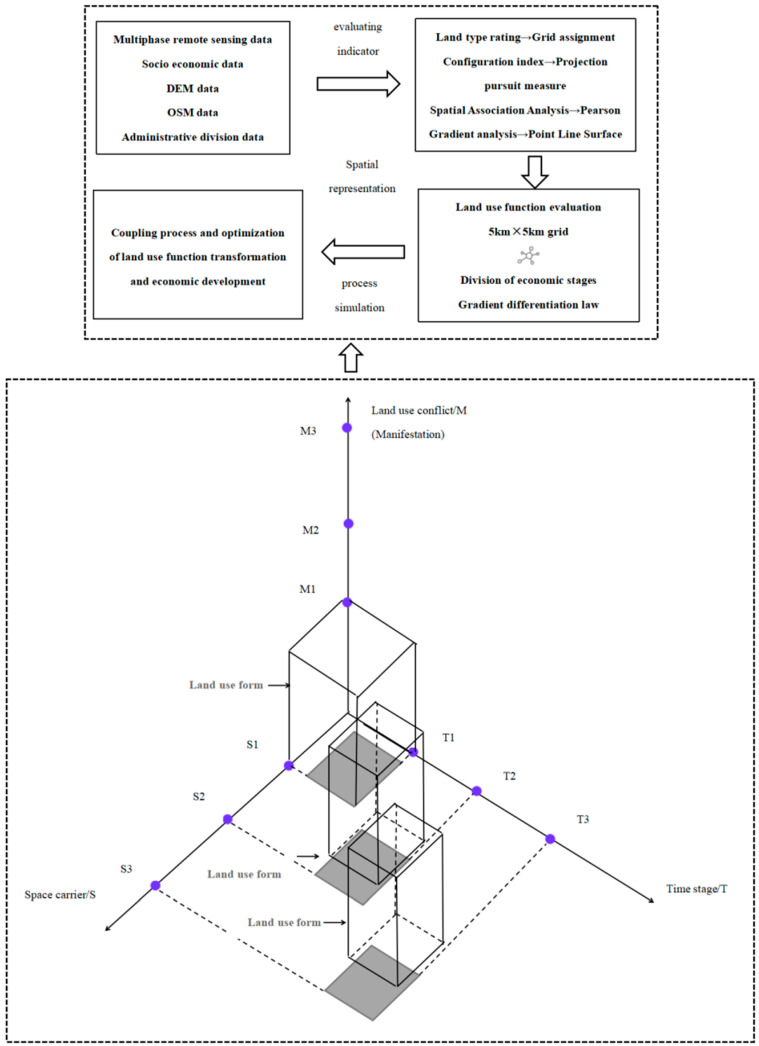
Workflow chart.

Spatial and temporal changes of land use in the Yangtze River Delta were measured from the grid scale, supported by GIS spatial analysis techniques. Moreover, we explore the coupling relationship between multifunctional land use and economic development levels in different regions through correlation analysis and the sample band method. Moreover, we explore the coupling relationship between multifunctional land use and economic development levels in different regions through correlation analysis and the sample band method. The specific workflow chart is shown in Figure 2.

#### 2.3.1. Multifunctional Land Use Measurement

Land use multifunctionality is both an inherent property of land and a process of social practice, where innate naturalness and sociality coexist. Researchers can only analyze land use functions at the natural and social levels if the study results have an integrated meaning [14]. Therefore, we used the current land use status as the basis and land use intensity as the grading criterion. Based on literature analysis [43,44], we consulted six professors in land use, rural geography, and ecology to rate the land use functions of different land classes. Ultimately, this study classified the various types of sites into seven classes based on their functional strength and positive and negative integrity. Take the production function as an example, strong production function (3 points), semi-production function (2 points), weak production function (1 point), and lack of function (0 points).

Furthermore, we considered the negative function of some sites (e.g., the negative effect of inland mudflats on the production function). We further divided the negative function into a robust negative production function (−3 points), a semi-negative production function (−2 points), and a weak negative production function (−1 point), compared to the positive function. (−1 point).

According to the SENSOR project’s “economic-social-ecological” system, we can divide land use into three main functions, such as production, living and ecological functions. However, as the problem of arable land protection and food security in China is always serious, we also draw on relevant scholarly studies [45,46,47] to split the production function into agricultural production and non-farm production function, for the purpose of farm-oriented and serving rural revitalization. This will also facilitate a better connection with the three types of space (agricultural space, urban space, and ecological space) delineated in the National Territorial Planning Outline (2016~2030).

Based on this, this study collated and scored the land use types in the Yangtze River Delta with specific criteria in the literature [43]. We then spatialized the land use function index using a 5 × 5 km cell grid. The sampling method was the equally spaced systematic method, generating a total of 15,169 cells so that each cell contains more than one land use type, which could simultaneously portray global characteristics and highlight spatial divergence and local change trends.

#### 2.3.2. Economic Gradient Division

This study classified economic gradients based on the hierarchical spatial distribution of economic development levels [48]. The usual methods to measure the level of economic development include the single-indicator method and comprehensive indicator method [49,50], such as using GDP per capita as a single measure. However, GDP per capita does not consider temporality and tends to underestimate or overestimate the level of economic development [48]. Compared with the single-biased evaluation, the comprehensive index method can cover industrial development, spatial structure, total station, and development trend [51]. However, the multiple covariances of indicators and weight sets are challenging [52]. With dimensionality reduction, the projection tracing method has a tremendous advantage in processing high-dimensional, nonlinear, and non-normal data. It also circumvents the subjectivity of weight setting [53]. There were many successful applications in arable land intensive use evaluation [54] and land use carrying capacity [55]. Thus, this paper draws on the results of previous studies [56,57] and considers the principles of regional characteristics and scientificity. We selected eight indicators, including GDP per capita, GDP per land, per capita total social fixed asset investment, per capita local fiscal revenue, total retail sales of consumer goods, non-agricultural share, disposable income of urban residents, and per capita net income of rural residents. We then combined the projection tracing method to measure the economic development level of each region in the Yangtze River Delta.

#### 2.3.3. Spatial Association Analysis

The doctrine of land use transition holds that the stage of economic and social development over some time always corresponds to the regional land use pattern, and the interaction between the two contributes to the coupled effect of land use transition and regional transition [11]. Based on the correlation coefficient to quantitatively measure the association between the two, it can effectively evaluate the correlation effect relationship between the two in long time series, which is both dynamic and systematic compared with the cluster analysis that relies on cross-sectional time points [58]. Under the condition that the two groups of random variables conform to the normal distribution, Pearson correlation analysis is more accurate and stricter in expressing the trend direction and intensity of their changes. Therefore, in this paper, firstly, the Kolmogorov–Smirnov regular distribution test (K-S test for short) was conducted on the land use multifunctional index and economic development level, and Pk-s >0.05 was considered that the data conformed to the typical distribution result. Otherwise, the study transformed the data with the BOX-COX method to conform to the specific distribution results. After normalizing the data, we used the Person correlation coefficient to characterize the correlation between the land use multifunctional index and the level of economic development. Finally, after the significance test, the absolute value of the correlation coefficient represents the strength of their correlation, and the positive and negative correlation coefficients represent the direction of their correlation.

## 3. Analysis of Results

### 3.1. Spatial and Temporal Evolution Characteristics of Multifunctional Land Use in the Yangtze River Delta

The spatial distribution and temporal evolution of the multifunctional land use index in the Yangtze River Delta from 2000 to 2020 have significant differences, while the spatial pattern represents a certain period of socio-economic development.

#### 3.1.1. Descriptive Statistics and Trend Analysis of Changes in 4 Functions

From 2000 to 2020, the average values of the agricultural production function, non-agricultural production function, living function, and ecological function in the Yangtze River Delta are constantly changing. Among them, the non-agricultural production and living functions gradually increase, and the agricultural production function and ecological function keep decreasing (Table 1). The proportional status of the function (contribution margin) reflects the strength and weaknesses of each function and its development status. Cross-sectionally, the contribution rate of the four function types during the study period was ranked as follows: agricultural production function > ecological function > living function > non-farm production function. The differences among the functions are significant, but there was a slight narrowing trend. Longitudinally, the most dramatic trend is in the non-farm production function, with a rate of change of 10.18% over the study period. The rate of change in the subsistence function was 2.71%, while the agricultural production and ecological functions decreased slightly in relative terms (−0.22% and −0.37%, respectively). The development of these four functions has been uneven and stage-specific. From 2000 to 2010, there was a rapid change in each function, followed by a slowdown. In conclusion, the Yangtze River Delta region’s agricultural production and ecological functions continue to weaken from 2000 to 2020, while the non-agricultural production and living functions continue to increase rapidly. Although agricultural production and ecological functions dominate, the uneven development of the functions and the stage characteristics need attention.

#### 3.1.2. Spatial and Temporal Evolution Characteristics of Agricultural Production Functions

**Figure 3:**
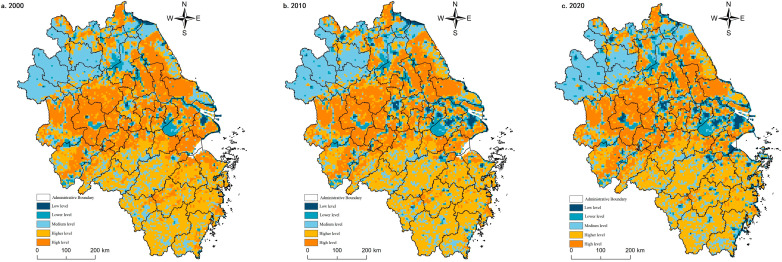
Spatial pattern of agricultural production functions in the Yangtze River Delta region, 2000–2020.

The agricultural production function in the Yangtze River Delta region was vital during the study period. However, the non-agricultural conversion of arable land caused by the coercion of construction land expansion [59] showed a decreasing trend year by year. The agricultural production function around the built-up area and the core area of the Shanghai metropolitan area decreased significantly. It was spatially convex in the middle and concave on both sides along the line of Lu’an-Chuzhou-Nantong City, with a gradient feature. The high-value areas were mainly located in the central Jianghuai Plain and the Lixia River Plain areas, with low-value concentrations around the built-up areas, the Yangtze River, the Huai River, Taihu Lake, Hongze Lake, and Chaohu Lake. Medium and lower-value areas were distributed discretely, mainly located in Fuyang and Bozhou in northern Anhui and the mountainous areas of southern Anhui, and the valley bottoms of the mountains in southwest Zhejiang. Low-value areas were distributed in point clusters in metropolitan areas, such as Shanghai, Nanjing, and Hangzhou (Figure 3).

In 2000, the proportion of high-value areas of agricultural production function was 20.78%, with stripes and blocks clustered in the Jianghuai Plain, the Lixia River Plain, and the Yellow Huaihai Plain. In 2010, the agricultural production function weakened, and the high-value and higher-value areas expanded mainly in the Yellow Huai and Plain areas in the north (e.g., Huai’an, Suqian). The middle area was distributed in southwest Zhejiang, north Anhui, and north Jiangsu coast. Low-value and lower-value areas expand in scope and are circled and progressive around urban built-up areas, Taihu Lake and Hongze Lake. In 2020, agricultural production functions continued to weaken, and high-value and higher-value areas were more stable, with little change in distribution scope and unit share. Medium areas increase mainly in south Anhui and south Zhejiang mountain areas, and low-value and lower-value areas expand in Hefei and Hangjiahu Plain.

The agricultural production function results from the local conditions and the situation. The agricultural production function is higher in areas with low topography, good soil, water, light, and heat configuration, continuous distribution of arable land, and developed farming civilization. In comparison, the agricultural production function is correspondingly lower due to natural constraints of topography (mountainous areas), geology (e.g., saline lands), and the duress of urbanization development [60]. However, the agricultural production function in the mountainous areas of southwest Zhejiang and southern Anhui has improved during the study period, indicating that the natural resource environmental constraints on agricultural production have decayed. The future agricultural production will be more diversified, intelligent, and green, and multifunctional agriculture and sizeable agricultural production oriented to market demand become the direction of transformation.

#### 3.1.3. Spatial and Temporal Evolution Characteristics of Non-Farm Production Functions

**Figure 4:**
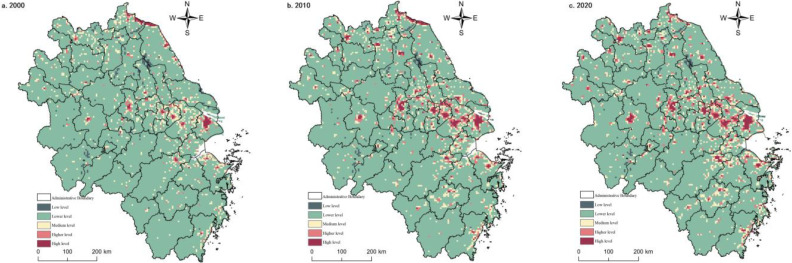
Spatial pattern of non-agricultural production functions in the Yangtze River Delta region, 2000–2020.

From 2000 to 2020, the non-agricultural production function grew gradually due to urban and rural construction land expansion. The non-agricultural production functions were higher in areas with higher levels of urbanization and industrialization. In Shanghai metropolitan area, Suzhou-Wuxi-Changzhou region, Hangzhou-Jiaxing-Hu plain, Nanjing city, Hefei city, and other places, network-type strengthening was present. Specifically, in 2000, high value and higher value areas accounted for only 2.64%, sporadically distributed in Shanghai, Nanjing, Hangzhou and Suzhou, Wuxi and Chang, and other central cities and circled decay. In 2010, the non-agricultural production function strengthened rapidly, and the average value of the whole region quickly increased, with a change rate of 14.12%. The percentage of high-value and higher-value areas grew to 5.25%. However, they were still concentrated around a few central cities (e.g., Shanghai, Nanjing and Hangzhou, Suzhou, Wuxi, and Changzhou areas, Hefei, Xuzhou, and Ningbo), and the regional gap had increased. The middle region ring high-value area expanded, and the change was relatively stable, with a proportion of 6.27% to 7.61%. Low-value and lower-value areas shrunk by 3.94% compared with the balance in 2000, primarily concentrated in northern Anhui, southwest Zhejiang, and Jiangsu north. In 2020, the non-agricultural production function was further enhanced, but at a relatively slower rate, with a change rate of 2.64%. The total percentage of high-value and higher-value zones increased by 0.83%, the same as in 2010. At this stage, spatial connectivity was enhanced, and the development of regional integration was apparent. 8.69% of the map units belong to the middle zone, a slight increase from 2010, mainly in the form of the surface surrounding the high-value site and accompanied by point clusters scattered throughout the region. The proportion of low-value and lower-value areas decreased by 1.93% compared with 2010, and the spatial distribution range did not change much (Figure 4).

Non-farm production functions depend on accumulating labor, capital, technology, and resources to produce scale effects. The circle diffusion effect arose with the strengthening of operations and increased scale. The added growth nodes could lead to synergies and network effects. Finally, the above results would continue to promote the growth and development of non-agricultural production functions. However, the asymmetric flow of factor resources also had spatial polarization and spatial stickiness, and the road strength dependence also locked regional differences due to development inertia [61,62,63,64].

#### 3.1.4. Spatio-Temporal Evolutionary Characteristics of Living Functions

**Figure 5:**
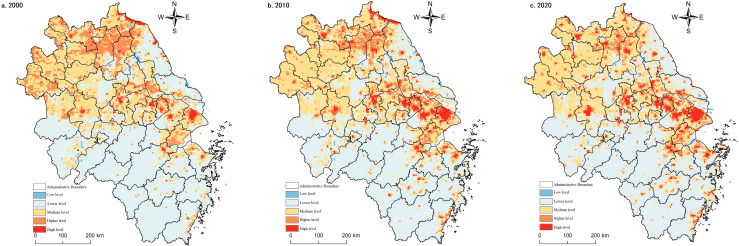
Spatial pattern of living functions in the Yangtze River Delta from 2000 to 2020.

The living functions of the Yangtze River Delta region were low during the study period, with an overall high in the north and a low in the south. This spatial pattern may be related to the fact that the north is plain and the south is more hilly and mountainous, thus influencing the regional distribution pattern of rural settlements. In general, the high-value and higher-value area of living function continued to expand during the study period. The spatial distribution was similar to the characteristics of the high-value and higher-value area of the non-agricultural production function. However, southern Jiangsu’s contiguous spread of living functions was more pronounced. At the same time, Suqian and Huai’an in the northern region and the coastal living functions in southeastern Zhejiang have also grown, mainly due to the relatively concentrated rural settlements. Specifically, the high-value and higher-value areas in 2000 are more focused in Shanghai, Suzhou-Wuxi-Changzhou, and Huai’an and Suqian cities in northern Jiangsu, with an area share of 5.46%. The medium-value areas are mainly distributed north of Anhui plain, accounting for up to 19.75%. However, the living functions of these areas are yet to be improved because the public infrastructure and public services in urban and rural areas are not perfect. Low-value and lower-value areas were mainly distributed in southern Anhui’s mountainous regions, southern Zhejiang’s central mountainous areas, and the hilly areas of the middle mountains of western Zhejiang, with an area share of up to 74.80%. These areas are widely distributed with forest, grassland, and other ecological lands and have large topographic undulations and geological disasters. In 2010, the high-value and higher-value areas changed faster, and the north-south divergence persisted. Shanghai and Suzhou-Xichang areas were visible as apparent intensification, and the area share was raised by 1.17%. The middle area had different clusters in Fuyang, Bozhou, Suizhou, Huaibei, and Bengbu in northern Anhui, and the proportion shrunk to 16.20%. The spatial distribution of lower-value and low-value areas is stable, and the proportion is still high (77.17%). In 2020, the high-value and higher-value areas expanded again, and the area share rose to 7.28%. The continuous trend of the core area of the Yangtze River Delta was highlighted, and the point-like strengthening of the northern part of Jiangsu Province (Xuzhou, Huai’an, Suqian) was gradually revealed. The medium zone has mostly stayed the same as the previous year and was still concentrated in northern Anhui. The range of low-value areas and low-value zones expanded, and the proportion increased to 78.46%. The living functions around ecological lands, such as water, woodland, and grassland, were reduced significantly (Figure 5).

The spatial and temporal differentiation of living functions is profoundly influenced by regional social and economic development, along with rapid urbanization and industrialization, the rapid expansion of urban and rural construction land, further massive concentration of population in a few cities and regions, more and more intense disturbance to the natural environment, the prominent contradiction between people and land, and increased natural environmental constraints.

#### 3.1.5. Spatial and Temporal Evolutionary Characteristics of Ecological Functions

**Figure 6:**
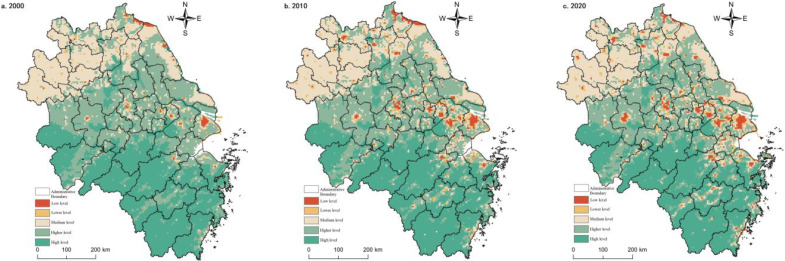
Spatial pattern of ecological functions in the Yangtze River Delta region from 2000 to 2020.

The ecological function of the Yangtze River Delta region is overall better but with a weakening trend during the study period. Spatially, the high south and low north distribution, such as the Dabie mountainous area, the mountainous area of south Anhui, the hilly area of west Zhejiang, the mountainous area of south Zhejiang, the ring of Taihu Lake, Hongze Lake, the coastal area of north Jiangsu and the estuary of Yangtze River had the most apparent decreasing trend. This phenomenon indicated that urbanization had a more substantial effect on ecological and environmental stress. In 2000, the proportion of high and higher value areas reached 46.41%, with a cluster-like appearance. In 2000, 46.41% of the high and higher-value regions were clustered in Zhejiang, southern Anhui, and central Jiangsu. The medium-value areas were more extensive and widespread, mainly in the northern Anhui and Jiangsu clusters. The lower-value and lower-value areas were scattered in Shanghai, Suzhou, Xichang, and Nanjing sporadically, with an area of 22.89%. In 2010, the higher-value and higher-value regions did not change much compared to the previous year, and their distribution was roughly consistent with the ecological land area. The medium-value areas shrank by 6.61%, mainly due to the expansion of central cities such as Shanghai, Nanjing, Hangzhou and Suzhou, Wuxi, and Changzhou to the periphery. The area share of low-value and lower-value areas increased by 2.2%, consistent with the central city expansion range. In 2020, the ecological function of the Yangtze River Delta region continued to decline, maintaining a medium level overall, and regional differences are still prominent, with high-value and higher-value areas locked in the core areas of mountains and hills, rivers, and lakes. However, the area share was compressed to 48.95% due to increased human activities. The middle zone did not change much, but the low-value and lower-value zones expanded near the central city, north of Anhui and north of Jiangsu (Figure 6). The strength of ecological functions was very dependent on the natural background environment. However, the increase in human activities, intensity, and disturbance inevitably brought a series of ecological and environmental problems, so the gradually increasing social and human factors of coercion should be addressed.

### 3.2. Evolution of Functional Synergies and Trade-Offs in Land Use

#### 3.2.1. Characteristics of the Temporal Evolution of the Synergy/Trade-Off Relationship of Land Use Functions

**Figure 7:**
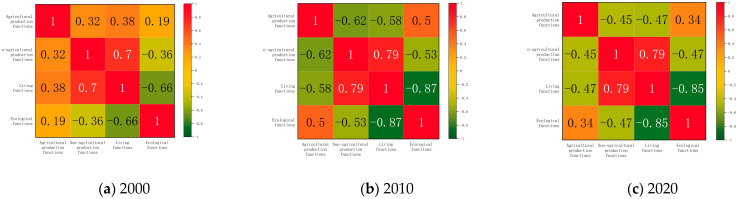
Person-related characteristics of land use functions in the Yangtze River Delta region, 2000–2020.

Spatial heterogeneity existed among the four functions in the Yangtze River Delta region, and the overlapping and competing coexistence of functions would result in concurrent synergy/balance relationships [65], which would increase multifunctionality and reduce trade-offs for sustainable regional development. This study diagnosed the synergistic/balancing relationships between functions and their degree of action based on the correlation coefficients’ positive and negative absolute magnitude. Precisely, the agricultural production function and non-agricultural production had a gradually increasing balance relationship, and the correlation coefficient decreased sharply by 0.77 during the study period. This phenomenon was due to the uncontrolled expansion of urban and rural construction land encroaching on agricultural lands, such as arable land, grassland, and garden land. Thus, the agricultural production function declined while non-agricultural production increased rapidly. The agricultural production function also had a strong trade-off relationship with the subsistence function, and the negative correlation strengthened significantly and accelerated. In contrast, the agricultural production function had a weak positive correlation with the ecological function, with a smooth trend of synergistic development between the two. This phenomenon could indicate that arable and garden ecosystems provide stable biological products such as food and fruits for humans while providing new semi-natural habitats. The story of non-agricultural production and living functions were closely related and had always shown a strong positive correlation during the study period, but the synergistic relationship between them decreased slightly in the more rapid development stage, and the human-centered development approached and orientation still need attention. The trade-off relationship between non-agricultural production and ecological functions fluctuated and increased to a peak (−0.53) from 2000 to 2010, and with the implementation of policies, such as environmental civilization construction, the negative relationship between them with the performance of procedures, such as the construction of ecological civilization, the negative correlation between the two had become weaker. The trade-off between living and ecological functions has been increasing, and the intensity of human activities in the Yangtze River Delta region has inevitably disturbed important ecological land, making the environmental service function weaker (Figure 7). In conclusion, agricultural production had a strong trade-off relationship with non-agricultural production and living functions, and agricultural production and ecological functions developed synergistically in general. In contrast, the synergy effect between non-agricultural production and living functions gradually increased, and there was a trade-off between non-agricultural production and living functions and ecological functions.

#### 3.2.2. Spatial Characteristics of Land Use Functional Synergy/Trade-Off Relationships

The dynamic rate and direction of each image element’s temporal evolution of land use functions were inconsistent. The interaction relationships of land use functions of individual image elements differed [66]. Therefore, we continued to call the corrcoef process in MATLAB to discriminate the interactions of land use functions of each image element in the study period. After significance tests (*p* < 0.05), the proportion of synergistic image elements weighed and not significantly correlated was counted in this study. The balance of trade-off image elements of agricultural production function-nonagricultural production function reached 78.88%, indicating that the spatial trade-off relationship between the two was dominant, and the spatial synergistic relationship was sporadically distributed in Huai’an in northern Jiangsu Province and along the Yangtze River (Anqing, Tongling, southern Nanjing), and the proportion of non-significant relationship was small. The spatial relationship characteristics of agricultural production-living function were similar to those of non-agricultural production but had a synergistic relationship in the central region. The main reason for this was the hollowing out and marginalization of the countryside, and the decreasing attractiveness of agricultural production to the population. Agricultural production-ecological functions were dominated by spatial synergistic relationships (89.39%), and local trade-off relationships appear within the scope of large ecological patches, water-covering areas, and river ecological corridors, such as the Dabie Mountains and Taihu Lake. The non-agricultural production-living function was dominated by a spatial synergistic relationship, with 79.70% of the image elements showing a synergistic relationship, scattered in clusters in Zhejiang, southern Jiangsu, etc. The trade-off relationship was interspersed in Anhui (e.g., Huangshan, Xuancheng, Suzhou) and northern Jiangsu (e.g., Yancheng, Suqian), with no significant association in space. The spatial trade-off between non-agricultural production and ecological functions dominated (87.99%), and insignificant synergistic relationships were only scattered in the point clusters of Huai’an, Changzhou, and southern Nanjing. The spatial trade-off between living and ecological functions dominates, with the absolute dominance of living functions and the relative weakening of environmental parts, indicating that the ecological environment was damaged in the process of socio-economic development. The proportion was not high, gathered in a mosaic shape in Huai’an, Suqian.

### 3.3. Economic Gradient Analysis of the Spatial and Temporal Evolution of Land Use Functions

Regional land use and economic development influenced and promoted each other, and the economic development stage impacted the industrial structure and social groups’ goals. The demand for land supply was different, resulting in other features of land use function evolution. In rapid urbanization, the dynamic relationship between “economy-society-ecology” was prone to imbalance. Therefore, clarifying the characteristics of the relationship between the growth of land use function and economic development was conducive to formulating adaptive land spatial management policies.

#### 3.3.1. Economic Development Gradient Division

This study used the projection tracing method to comprehensively assess the economic development level of 41 municipalities in the Yangtze River Delta through the measurement of seven socioeconomic indicators. We then highlighted cluster homogeneity and variability with the natural break method to discover the data distribution patterns.

**Figure 8:**
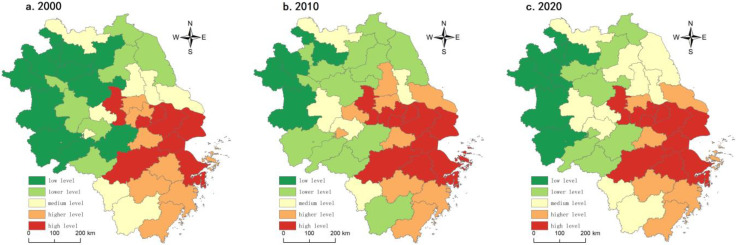
Economic development level of the Yangtze River Delta region, 2000–2020.

From 2000 to 2020, the coefficient of variation and the extreme difference in the economic development level of the Yangtze River Delta fluctuated slightly. However, it always maintained a high level, with the mean values of the coefficient of variation and the extreme difference being 0.6412 and 2.220, respectively, indicating that the regional economic development level was polarized, spatially unbalanced, and geographically different. Specifically, in 2000, there were 11 municipalities at low economic development levels, widely concentrated in northern and western Anhui. Seven towns were at a lower level of economic development, mainly distributed in two major clusters north of Jiangsu Province and the Jianghuai region of Anhui Province. Eight medium-level municipalities were primarily distributed in central Jiangsu and southeastern Zhejiang. There were eight municipalities in the higher level name, mainly distributed in Zhenjiang, Huzhou, Shaoxing, Jinhua, Taizhou, and other places. The high-level areas were primarily concentrated in Shanghai, Nanjing, Hangzhou, Suzhou, Wuxi, and Changzhou. In 2010, the overall economic level tended to be better. The number of low-level units decreased to four, mainly in Bozhou, Suizhou, Fuyang, and Luan in northern and western Anhui. These places had an outflow of population, low industrial structure, far from regional growth poles, and a lack of internal and external power pull for economic development. The lower unit level had increased, with a total of 13, mainly in southern Anhui, eastern Anhui, and northern Jiangsu mountain areas. These areas were either constrained by deviations in natural factor endowments (undulating terrain), unsatisfactory location conditions (lack of transportation routes), or regional development strategies that did not focus on economic development: five medium-level municipalities, mainly Xuzhou, Hefei, Taizhou, Wuhu, and Quzhou. However, there were lagging effects, such as capital investment and industrial restructuring, so continuously consolidating the development effect was still necessary. In 2020, the economic development level would be stable, but the regional differences were still enormous. Seven cities with low economic development would be concentrated in the northern Anhui and Dabie Mountains contiguously and fall into the “poverty trap”. Lower-level areas decreased to 8, primarily located in the mountainous regions of southern Anhui, the junction of Anhui and Jiangsu, and northern Jiangsu, such as Huangshan, Chuzhou, Suqian, and Lianyungang. The medium level increased significantly to 10 units, mainly located in central Jiangsu, Hefei-Bengbu, Xuzhou, Quzhou, and Lishui. Higher and high-level areas remained stable, mainly in Hangzhou, Jiahu, Suzhou, Xichang, Nanjing, and Shanghai (Figure 8).

Overall, the Yangtze River Delta region had a balanced number of high and low levels of economic development during the study period. However, the dynamic evolution process had a path-locking effect, and regional differences persist, with a spatial pattern of high in the southeast and low in the northwest.

#### 3.3.2. Correlation Analysis

The primary research object of the Outline of the Yangtze River Delta Regional Integrated Development Plan is the municipal administrative unit. Therefore, to reflect the precise changes in the evolution of land use function matching the stage of economic development and highlighting spatial heterogeneity and continuity, first, we used the regional analysis tool of ArcGIS software to transform the scale of micro land use function evaluation results into municipal administrative units. We then continued to observe the change characteristics of correlation coefficients between land use function indices and economic development levels in the Yangtze River Delta region in typical sample zones during the study period. Such a method can better express the numerical correlation and spatial interaction between the two. See Table 2.

Global correlation: Agricultural production function had a weak negative correlation with the level of economic development, indicating that agricultural production and socio-economic benefits were not synergistic, and due to the difference in comparative interests affecting farmers’ willingness to plant, it was easy to induce the non-agriculturalization of arable land and the non-foodization of arable land. Non-agricultural production function had a significant positive correlation with economic development, and the Yangtze River Delta region was the region with the most active economic growth, the highest degree of openness, and the most substantial innovation capacity in China. One of the regions with high proportion of secondary and tertiary production, complete industrial chain, advanced technology, talent reserve, and non-agricultural industries would still develop at a high speed in the future. According to the theory of environmental Kuznets curve (EKC) relationship, it is necessary to pay attention to the marginal effect of non-agricultural production and the quality of development, and change the mode of economic growth [67]. The positive impetus of living function on the level of economic development gradually strengthens, and living and working in peace and contentment make the population more highly concentrated. The ecological function had a weak negative correlation with the level of economic development but gradually decreased, and thanks to the strict control of construction land expansion and ecological green integration policies, the coercion of urbanization on the ecological environment was improving, which was consistent with the existing empirical findings [34,58]. The coercive effect of urbanization on the ecological environment was improving, which was consistent with the current empirical findings [40,68,69].

To more accurately discern the coupling between the dynamic evolutionary process of land use function and economic development, we calculated the mean values of land use function indices for different levels of economic growth in the Yangtze River Delta region during the study period. The results showed that in the progression of economic development from the low to high level, the agricultural production function grew steadily first. After the socioeconomic development, the contribution of agricultural production to economic development decreased. The non-agricultural production function, on the other hand, gradually increased. The index of the living function showed an irregular trend of decreasing, increasing with the level of economic development. The ecological function showed a rising–declining trend.

The above phenomena Indicated that the Yangtze River Delta region had substantial heterogeneity in terms of resource and environmental constraints, especially in the early stage of economic development, which may destroy the habitat environment. However, the quality of life would improve with the improvement of the governance level.

**Figure 9:**
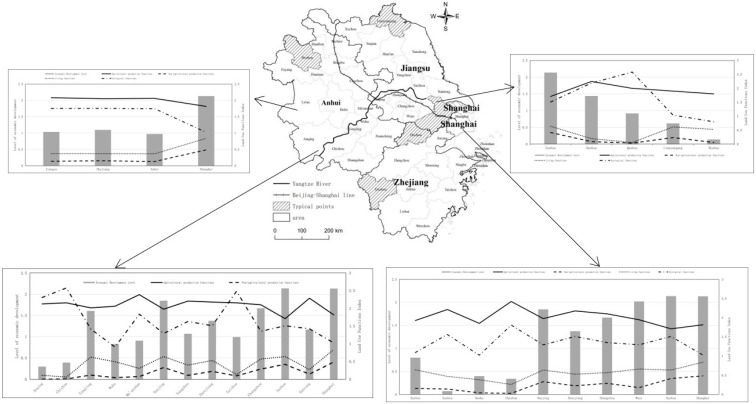
Distribution of land use function indices for different levels of economic development in the Yangtze River Delta region.

Gradient change: The evolution of land use function and regional economic and social development strongly coincided. For this reason, a typical sample belt in the Yangtze River Delta region was selected in a ‘point-line surface’ combination to analyze the spatial heterogeneity and variability of regional development in depth (Figure 9).

(1) Point: The economic development level of Suzhou, Huzhou, Quzhou, Lianyungang and Bozhou decreased in turn, and the change characteristics of land use function of the five towns were different, in general, the agricultural production function decreased with the increasing economic development level, but the regional agricultural production function index of Huzhou (higher economic development level) was the highest among the five towns, and the reason for this was that the agricultural industrialization in this place had a good development foundation, so it had become the main production area and essential production base in Zhejiang Province. The non-agricultural production function decreases with the level of economic development, but the non-agricultural production in Lianyungang (lower level of economic growth) changed abruptly, probably because it was a port city with a relatively large area of urban and rural construction land, and the ‘three new and one high’ industries, i.e., new medicine, new materials, new energy, and high-end equipment manufacturing, were developing rapidly. In the period of rapid economic development, the competition and conflict between functions would be more intense, and the spatial effect of functional balance would not threaten the living environment. The ecological function grew first and then decreased with the change in economic development level. The environmental function index was higher in Huzhou City and Quzhou, surrounded by mountains and water. However, the ecological function of Lianyungang and Bozhou, which were lagging in economic development, decreased in order, which indicated that the production-living function would highly threaten the ecological function when the economic development level is low. The trend of functional dispersion was apparent.

(2) Lines: Two main sample lines (along the Yangtze River and the Beijing-Shanghai high-speed railway line), which were horizontal and vertical from east to west and north to south, were selected, taking into account the continuity and heterogeneity of the geographical space, and the gradient of each city was well expressed. ① The agricultural production function along the Yangtze River was generally high in the west and low in the east. However, there were sharp change points (Maanshan and Nantong) and spatial jumps closely related to spatial suitability, regional policies, and land use subjects. The distribution and change patterns of the non-agricultural production and living function index were similar. Generally higher in the east and lower in the west, it was influenced by the development of urbanization. The function index was significantly higher in the regional central cities of Nanjing and Suzhou. The overall ecological function was high in the west and low in the east. However, the rhythm of change was different, and the changing pattern was irregular because the natural background environment influenced the ecological spatial design. ② The Beijing-Shanghai high-speed railway line runs north-south, spanning ten municipalities at different stages of economic development, with prominent differences in economic gradients. Agricultural production function tended to decrease from north to south, abruptly changing in Chuzhou, which was adjacent to the Nanjing metropolitan area. The non-farm production function and subsistence function index curves were nearly parallel and spatially distributed high in the south and low in the north. There needed to be more alignment between the level of economic development and ecological function in local units. However, there was a weak trend of elevated overall environmental function, indicating that economic growth did not necessarily coerce damage to ecological function. The influence of economic development methods was also significant.

(3) Surface: The geographical differences among the three provinces and one city were more prominent, and we continued to observe the coupling correlation between land use function and economic development level in each region from the provincial level. The results showed that agricultural and ecological production functions significantly decreased with higher economic development levels, and living and non-farm production functions increased dramatically with higher economic development levels.

Socioeconomic development influences land use functions, and sequential choices of land use functions trigger games of interest and spatial conflicts/mitigation in different sectors [42]. Generally, regional land use functions correspond to their economic and social development stages. However, the transformation of land use functions emphasizes the dynamic and regional nature [11]. So, different functions are spatially mutable and jumpy.

## 4. Discussion and Conclusions

### 4.1. Discussion

#### 4.1.1. Study Applicability

Land use function transformation follows the law of regional differentiation and socio-economic development. The study of trendy transitions of land use function patterns is a hot spot in the field [15,25,70,71]. Due to the different levels of economic development in different regions, the strong and weak primary and secondary choices of land use functions vary. The dominant functions, sequential options of functions, and inter-functional trade-off/synergy relationships differ in different development stages. The Yangtze River Delta region is one of China’s most socio-economically developed regions. However, its human–land relationship is tense, resource and environmental constraints are high, and internal geographical elements and economic development have noticeable gradient changes. With the integration of the Yangtze River Delta rising as a national development strategy in China, it is of theoretical and practical significance to pay attention to the transformation of land use functions in the Yangtze River Delta region and the coupling relationship between them and economic development.

This study focuses on the spatial characteristics of land use functional transformation, which is usually triggered by socio-economic development [7,8,9,10,11]. Therefore, this paper analyzes the gradient change characteristics of land use functional transformation in a ‘point-line surface’ approach, emphasizing the regional nature of land use transformation [9], and selects typical sample zones. This approach makes up for the weak empirical evidence and lack of spatiality of previous studies.

The land use function transition is a process of intrinsic economic sector interest trade-off and spatial game [25]. The land use function pattern, relationship, and effect vary at different stages of economic development [29,31]. This study shows that over the past 20 years, the land use function in the Yangtze River Delta region has demonstrated a gradient of ‘central city-peri-peri-peri-rural’. At different stages of economic development, the land use function extends outward in different ways, such as core diffusion, hierarchical diffusion, wave-like diffusion, and jump diffusion. Finally, it reveals discrete, aggregation, distribution, and balanced spatial structures. The pattern influences the process, and the process changes the way, thus checking and balancing socioeconomic development to drive the growth of land use functional transformation [72].

#### 4.1.2. Research Shortcomings and Outlook

Due to the complexity, nonlinearity, and ebb and flow of land systems, land use functions’ trade-off/synergy relationships are heterogeneous and dynamic. The induced spatial spillover effect and time lag will also be different. Previous studies have shown that there was a trade-off between tea production and ecological function in Anji County, Zhejiang Province, an economically developed region in eastern China, and that ecological function declined significantly with the expansion of tea plantation production [73]. Agricultural production in Zhangye, Northwest China, had a synergistic relationship with net primary productivity and a trade-off relationship with water availability/soil conservation [74]. The ecological and productive function trade-off/synergy relationship in Zhangjiakou City, an ecologically fragile area, alternately shifted from synergy to trade-off between environmental and livelihood functions [29]. The results of this paper showed that ecological functions compete intensely mainly with the production of basic livelihood security. In addition, there was a robust spatial trade-off between agricultural and non-agricultural production. Moreover, the ecological function synergizes with agricultural production for food supply. This showed significant spatial and temporal heterogeneity in the land use multifunctional trade-offs. However, the trade-offs between functions were not invariable. Even for the exact configuration of functional combinations, the trade-offs in different regions and levels of economic development have differentiated characteristics. This showed significant spatial and temporal heterogeneity in the land use multifunctional trade-offs. However, the trade-offs between functions were not invariable. Even for the exact configuration of functional combinations, the trade-offs in different regions and levels of economic development have differentiated characteristics. Therefore, we must focus on balanced spatial development and manage functional spatial zoning for food production, residential life, and ecological service supply. This showed significant spatial and temporal heterogeneity in the land use multifunctional trade-offs. However, the trade-offs between functions were not invariable. Even for the exact configuration of functional combinations, the trade-offs in different regions and levels of economic development have differentiated characteristics. Therefore, we must focus on balanced spatial development and manage functional spatial zoning for food production, residential life, and ecological service supply. In addition, the selection of land use function in this paper lacked attributes such as culture and subject perception. Moreover, the sample size of the sample zone unit selection was small, and the study also needed to be broadened in terms of period. We will increase the scope of the research and conduct a long time-series follow-up of the typical units in the subsequent study.

### 4.2. Conclusions

The study constructed a multi-functional evaluation index system of land use from the perspective of ‘production life ecology’. We then used the methods of Person correlation analysis and transect analysis. Furthermore, this paper discussed the spatial and temporal evolution and economic gradient differentiation of land use multi-function in the Yangtze River Delta from 2000 to 2020. This paper draws the following conclusions:

(1) There were apparent spatial heterogeneity and time-series dynamics in the Yangtze River Delta’s evolution of land use functions. The multifunctional land use index of the Yangtze River Delta region from 2000 to 2020 showed agricultural production function > ecological function > living function > ecological function. However, the agricultural and environmental functions continued to weaken, and the non-agricultural production and living functions kept increasing rapidly. Regarding spatial distribution, the agricultural production function was coerced by expanding construction land and converting arable land to non-agricultural use. The agricultural production function decreased yearly around the built-up area and in the core area of the Shanghai metropolitan area, with a gradual change along the line of ‘Luan-Chuzhou-Nantong’. The non-agricultural production function was more substantial in areas with higher urbanization and industrialization, Shanghai metropolitan area, Suzhou-Wuxi-Changzhou region, Hangzhou-Jiaxing-Hu plain, Nanjing, and Hefei showed a network-type reinforcement, and regional differences had strengthened. The spatial distribution was similar to that of the high-value and higher-value areas of non-agricultural production functions, and the spatial spread of the function in the southern part of Jiangsu Province was prominent. At the same time, the living function enhanced significantly in Suqian City and Huai’an City in the north and on the southeastern coast of Zhejiang. The overall ecological function was better but weakened and generally distributed high in the south and low in the north. The ecological function was under greater stress in the ecologically fragile and sensitive mountainous areas, waters, and inlets to the sea.
(2) The trade-off/synergistic relationships among land use functions in the Yangtze River Delta region from 2000 to 2020 were interspersed and varied significantly in space and time. By introducing the analysis method of ecological service trade-off/synergistic relationship, this paper revealed the functional interactions and spatial distribution more clearly: the trade-off relationship between agricultural production and non-agricultural production and living functions was dominant, and the spatial synergistic relationship was sporadically distributed in Huai’an in northern Jiangsu Province and along the Yangtze River (Anqing, Tongling, and southern Nanjing). The overall synergistic development between agricultural production and ecological functions, and the local trade-off relationship appeared in extensive ecological. The synergistic relationship between non-agricultural production and living was enhanced, and the trade-off relationship was interspersed in Anhui (Huangshan, Xuancheng, Suzhou, etc.) and northern Jiangsu (Yancheng, Suqian, etc.). The trade-off relationship between non-agricultural production, living function, and ecological function was dominant, and the synergistic non-significant relationship was only scattered in Huai’an, Changzhou and southern Nanjing in point clusters.
(3) The spatial and temporal evolution of land use functions in the Yangtze River Delta region from 2000 to 2020 was characterized by a significant economic gradient divergence. This study found the following results by Person correlation and sample band analysis: Agricultural production function had a weak negative correlation with economic development level. Non-agricultural production function, living function, and economic development had a significant positive correlation and gradually increased. Ecological function had a weak negative correlation with economic development level and steadily weakened. During the progression of economic development from low to the high level, the evolution trend of each function was different, with non-agricultural production steadily increasing, agricultural production and ecological function growing to a particular stage and then decreasing, and living function showing an irregular trend of decreasing–raising. Regional land use functions correspond to their economic and social development stages, but different functions were spatially mutable and jumpy.

## Figures and Tables

**Table 1:** Descriptive statistics and contribution rates of multifunctional land use in the Yangtze River Delta region, 2000–2020.

Year	Agricultural Production Function			Non-Agricultural Production Functions			Living Functions			Ecological Function		
	2000	2010	2020	2000	2010	2020	2000	2010	2020	2000	2010	2000
Average	2.14	2.07	2.03	0.05	0.12	0.15	0.25	0.34	0.38	1.93	1.82	1.77
Percentage	48.97	47.48	46.78	1.16	2.82	3.53	5.76	7.84	8.88	44.12	41.86	40.81

**Table 2:** Correlation between land use function index and economic development level.

	Agricultural Production Functions			Non-Agricultural Production Functions			Living Functions			Ecological Functions		
	2000	2010	2020	2000	2010	2020	2000	2010	2020	2000	2010	2020
Economic Development	0.047 *	−0.23 *	−0.28 *	0.53 *	0.55 *	0.57 *	0.036 *	0.39 *	0.31 *	0.079 *	−0.083 *	−0.021 *

Note: * indicates significant correlation at the 0.05 level (two-sided).

## Data Availability

No new data were created or analyzed in this study. Data sharing is not applicable to this article.
